# Brain and Spinal Cord Interaction: Protective Effects of Exercise Prior to Spinal Cord Injury

**DOI:** 10.1371/journal.pone.0032298

**Published:** 2012-02-22

**Authors:** Fernando Gomez-Pinilla, Zhe Ying, Yumei Zhuang

**Affiliations:** 1 Department of Integrative Biology and Physiology, University of California Los Angeles, Los Angeles, California, United States of America; 2 Department of Neurosurgery, UCLA Brain Injury Research Center, University of California Los Angeles, Los Angeles, California United States of America; Mental Health Research Institute and the University of Melbourne, Australia

## Abstract

We have investigated the effects of a spinal cord injury on the brain and spinal cord, and whether exercise provided before the injury could organize a protective reaction across the neuroaxis. Animals were exposed to 21 days of voluntary exercise, followed by a full spinal transection (T7–T9) and sacrificed two days later. Here we show that the effects of spinal cord injury go beyond the spinal cord itself and influence the molecular substrates of synaptic plasticity and learning in the brain. The injury reduced BDNF levels in the hippocampus in conjunction with the activated forms of p-synapsin I, p-CREB and p-CaMK II, while exercise prior to injury prevented these reductions. Similar effects of the injury were observed in the lumbar enlargement region of the spinal cord, where exercise prevented the reductions in BDNF, and p-CREB. Furthermore, the response of the hippocampus to the spinal lesion appeared to be coordinated to that of the spinal cord, as evidenced by corresponding injury-related changes in BDNF levels in the brain and spinal cord. These results provide an indication for the increased vulnerability of brain centers after spinal cord injury. These findings also imply that the level of chronic activity prior to a spinal cord injury could determine the level of sensory-motor and cognitive recovery following the injury. In particular, exercise prior to the injury onset appears to foster protective mechanisms in the brain and spinal cord.

## Introduction

Spinal cord injury (SCI) is a major cause of disability in civilian and military environments, and involves long-lasting hardships to victims and families. Besides the main deficits in motor and sensory functions, cognitive and emotional disorders are common features in patients suffering from SCI [1]. New evidence indicates that loss of spinal cord (SC) input to the brain can promote profound functional modifications in brain centers, such as rapid changes in the spontaneous electrophysiological activity of neuronal networks [2]. In addition, new research is starting to reveal that lesion to the spinal cord can impact molecular systems important for synaptic plasticity in the brain [3], [4]. Accordingly, we have initiated studies to determine whether a SCI can concert the action of molecular systems that regulate neuronal plasticity in the hippocampus and SC. We have focused our studies in the early injury period, as these events can have critical implications for long-term plasticity [5]. The results of these studies can provide critical information to start understanding the mechanisms by which SCI can affect an array of functions beyond motor disorders, and can provide new clues how to enhance functional recovery.

Exercise is considered beneficial for overall health of the organism during homeostatic and disease conditions. A growing body of evidence indicates that exercise provided after the onset of a SCI counteracts some of the effects of the lesion [6], [7]; however, the capacity of exercise when provided before the injury onset are not clearly established. Accordingly, we have embarked in studies to evaluate the protective effects of exercise in the brain and SC of rodents receiving a full transection to the SC. This information is significant to understand the potential for functional recovery of SCI patients based on their prior lifestyle history, and has direct applicability to evaluate prognosis in injured athletes.

Studies have been focused on molecular systems associated with the action of brain-derived neurotrophic factor (BDNF). BDNF is a powerful synaptic facilitator [8], and promotor of neuronal plasticity [9], [10], and its production is influenced by exercise in the hipocampus [11] and spinal cord [12]. Given that exercise has a strong influence on learning and memory ability associated with hippocampal BDNF [11], we focused this study on the hippocampus to evaluate potential effects of SCI on the substrates of cognitive function. We have assessed synapsin I and GAP-43 based on their involvement on the mechanisms by which BDNF affects neuronal and synaptic plasticity. Synapsin I is a well-characterized member of a family of nerve terminal-specific phosphoproteins and is implicated in neurotransmitter release, axonal elongation, and maintenance of synaptic contacts [13]. BDNF stimulates the synthesis and activation of synapsin I resulting in elevated neurotransmitter release [14]. GAP-43 has important roles in axonal growth, neurotransmitter release [15], [16], and learning and memory [17].

## Results

### Effects of SCI and exercise on the hippocampus

#### BDNF

**Figure 1:**
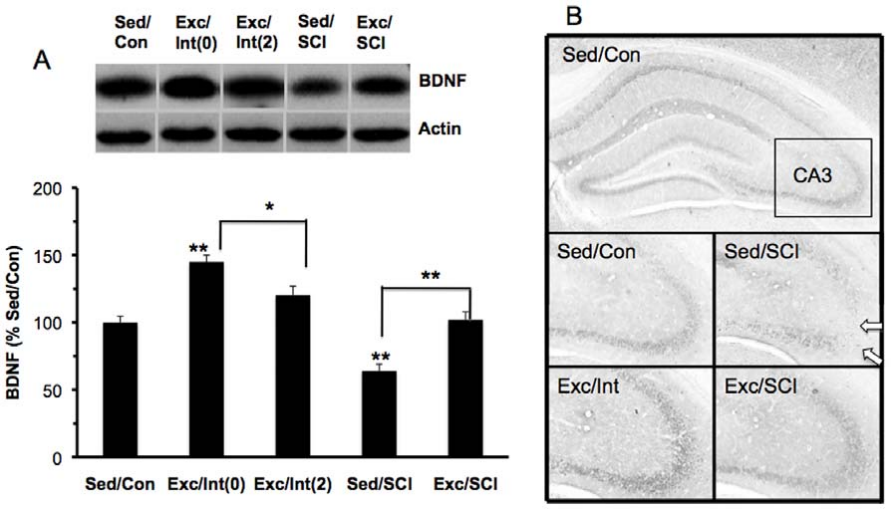
Effects of SCI and exercise on BDNF levels in the hippocampus. (A) Exercise elevated levels of BDNF in intact animals sacrificed on the last exercise day (Exc/Int0) or two days after ceasing exercise (Exc/Int2). SCI reduced BDNF level and exercise prior to SCI prevented this reduction. *p<0.05, **p<0.01. Representative western blot bands for BDNF are shown for each animal group. (B) Microphotographs of representative immunohistochemical sections showing localization of BDNF in the hippocampus. SCI reduced BDNF immunolabeling (white arrows, Sed/SCI) mainly affecting the CA 3 region, but exercise prior to the SCI prevent these effects (black arrows, Exc/SCI).

One-way ANOVA analysis showed a significant group effect (F(4,38) = 44.578, p<0.001) for the hippocampal levels of BDNF. We included two intact groups, Int(0) and Int(2), in our design to control for possible effects of the two-day resting period on BDNF levels. The Bonferroni post-hoc test indicated that exercise elevated levels of BDNF (143%, p<0.01) in the intact animals sacrificed immediately after the last exercise bout (Exc/Int(0), but promoted an increasing tendency (120% (p>0.05) in the animals sacrificed two days after exercise Exc/Int(2), compared to sedentary control animals (Sed/Con, 100%). With regards to the effects of spinal cord injury on the hippocampus, there was a reduction in BDNF level in the sedentary injured animals (Sed/SCI, 63%, p<0.01) while exercise prior to the injury (Exc/SCI, 103%) prevented this reduction, as compared to the sed/Con group. Levels of BDNF in the exercise animals exposed to SCI (Exc/SCI) showed a decreasing tendency (p>0.05) relative to Exc/Int(2) animals (Fig. 1A).

We performed immunohistochemistry for BDNF in order to evaluate the phenotypic expression of protein changes detected with western blot analysis. Qualitative analysis under light microscopy showed weak BDNF labeling in the various hippocampal subregions in sedentary controls, and the intensity of the labeling became more prominent along the CA3 and dentate gyrus (DG) (Fig. 1B). The SCI reduced the intensity of the BDNF labeling; however, exercise appeared to attenuate this reduction, as verified with the western blot analysis.

#### Synaptic markers (Fig. 2)

**Figure 2:**
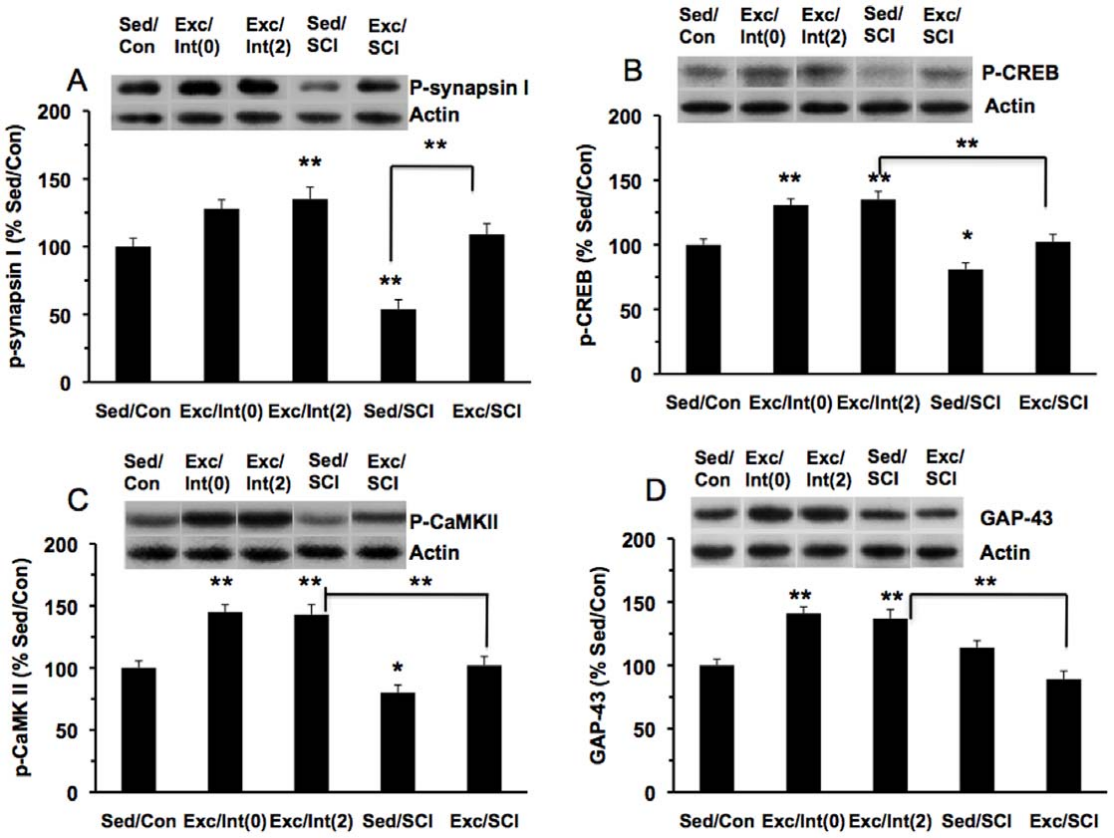
Effects of SCI and exercise on plasticity markers in the hippocampus: (A) p- synapsin I; (B) p-CREB; (C) p-CaMKII; (D) GAP-43. Exercise increased protein levels of these markers in intact animals sacrificed on the last exercise day (Exc/Int(0)) or two days after ceasing exercise (Exc/Int(2)). Exercise prior to SCI prevented the effects of the injury. *p<0.05, **p<0.01. Representative western blot bands are shown for each experimental group.

For the levels of p-synapsin I (Fig. 2A), there was a statistically significant difference between groups as determined by one-way ANOVA (F(4,38) = 22.929, p<0.01). The Bonferroni post-hoc test was used for multiple comparisons showed that exercise increased the p-synapsin I levels in the Exc/Int(0) group (124%, p>0.05). There was a significant increase (137%, p<0.05) in p-synapsin I levels in the Exc/Int(2) group. SCI significantly reduced (54%, p<0.01) the levels of p-synapsin I while exercise prior to the SCI counteracted these effects such that the p-synapsin I levels in the Exc/SCI group remained nearby the Sed/Con group (Fig. 2A).

With regards to p-CREB (Fig. 2B), one-way ANOVA showed that there was a significant group effect (F(4,38) = 23.112, p<0.01). The Bonferroni post-hoc test for multiple comparisons revealed that exercise increased p-CREB levels significantly in both intact groups [Exc/Int(0): 131%, p<0.01; Exc/Int(2): 137%, p<0.01]. SCI reduced the levels of p-CREB in sedentary animals (Sed/SCI: 79%, p<0.05) while exercise prevented this decrease such that p-CREB remained nearby the control levels (Exc/SCI: 102%). Furthermore, p-CREB levels of Exc/SCI group were significantly lower than Exc/Int(2) group (137% vs. 102%, p<0.01; Fig. 2B).

With regards to p- CaMKII, one-way ANOVA showed a significant group effect (F(4, 38) = 25.086, p<0.01; Fig. 2C). The Bonferroni post-hoc test for multiple comparisons indicated that exercise increased the p-CaMKII levels in the Exc/Int(0) group (144%, p<0.01) and the Exc/Int(2) group (143%, p<0.01), as compared to Sed/Con group (Fig. 2C). SCI reduced the levels of p-CaMKII in the Sed/SCI group (79%, p<0.05) as compared to the Sed/Con group, while exercise prevented these reductions (Exc/SCI: 99%, Fig. 2C). SCI counteracted the effects of exercise as compared to Exc/Int(2).

With regards to GAP-43, one-way ANOVA analysis showed a significant group effect (F(4,38) = 16.077, p<0.01). The Bonferroni post-hoc test for multiple comparisons showed that exercise increased the levels of GAP-43 in both Exc/Int(0) and Exc/Int(2) groups (140% and 136% respectively, p<0.01), as compared to the Sed/Con group. There was an increasing tendency in the Sed/SCI group (116%, p>0.05). SCI did not alter the levels of GAP-43 in exercise animals (Exc/SCI: 89%, p>0.05), as compared to Sed/Con (Fig. 2D); however the level of GAP-43 were decreased in exercise animals with SCI (Exc/SCI) as compared to Exc/Int(2) animals (p<0.01) (Fig. 2D).

### Effects of SCI and exercise on the spinal cord

#### BDNF

**Figure 3:**
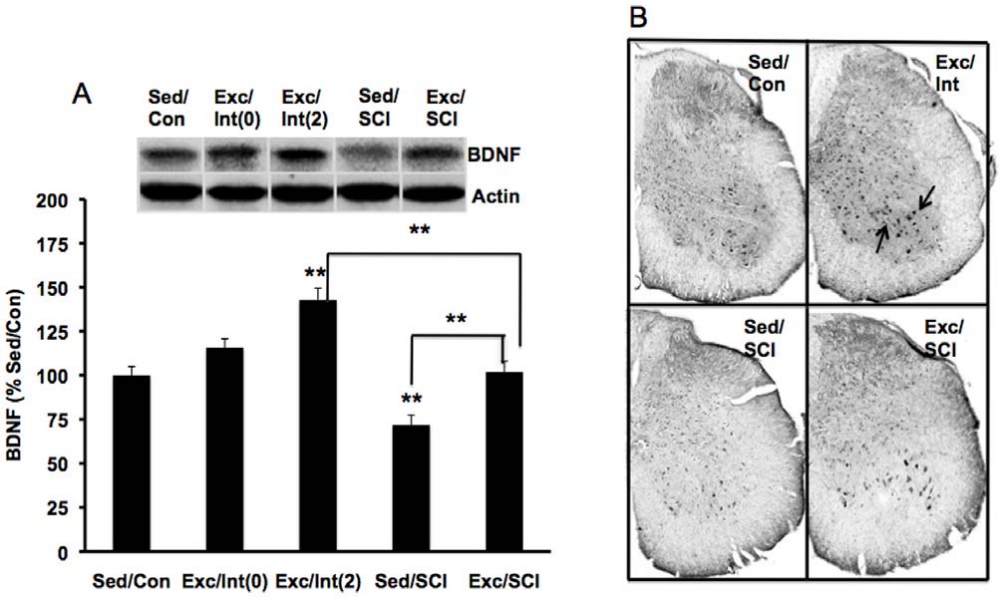
Effects of SCI and exercise on BDNF levels in the lumbar region of the spinal cord. (A) Exercise elevated levels of BDNF in intact animals sacrificed on the last day of exercise (Exc/Int(0)) or two days after ceasing exercise (Exc/Int(2)). SCI reduced levels of BDNF while exercise counteracted these effects (Exc/SCI). Representative western blot bands displaying BDNF are shown for each animal group. **P<0.01. (B)Microphotographs of representative immunohistochemical sections showing localization of BDNF in the hippocampus. SCI reduced BDNF immunolabeling (Sed/SCI) mainly affecting motoneurons (white arrows), but exercise prior to the SCI prevented these effects (black arrows; Exc/SCI).

To evaluate the effects of exercise prior to SCI in the spinal cord, we measured BDNF protein levels in the lumbar enlargement region (Fig. 3). One-way ANOVA showed a significant group differences (F(4, 38) = 27.024, p<0.01). The Bonferroni post hoc test showed that BDNF levels were increased in the Exc/Int(2) group (143%, p<0.01) while there was an increasing tendency (116%, p>0.05) in Exc/Int(0), compared to the Sed/Con group (Fig. 3A). With regards to the effects of the injury, SCI reduced BDNF levels to 72% (p<0.01) of Sed/Con in the Sed/SCI group, while exercise prior to the SCI (Exc/SCI) counteracted this reduction (Fig. 3A).

We performed BDNF immunohistochemistry in the SC lumbar enlargement region in order to illustrate the phenotypic expression for changes in proteins levels quantified using western blot analysis. Qualitative analysis using light microscopy showed that BDNF was localized to motoneurons in the ventral horn and their axonal processes coursing through the white matter. There was a relative decrease in BDNF immunostaining in the SC of lesioned mice (Fig. 3B) while exercise appeared to preserve the immunostaining (Fig. 3B).

#### Synaptic markers

**Figure 4:**
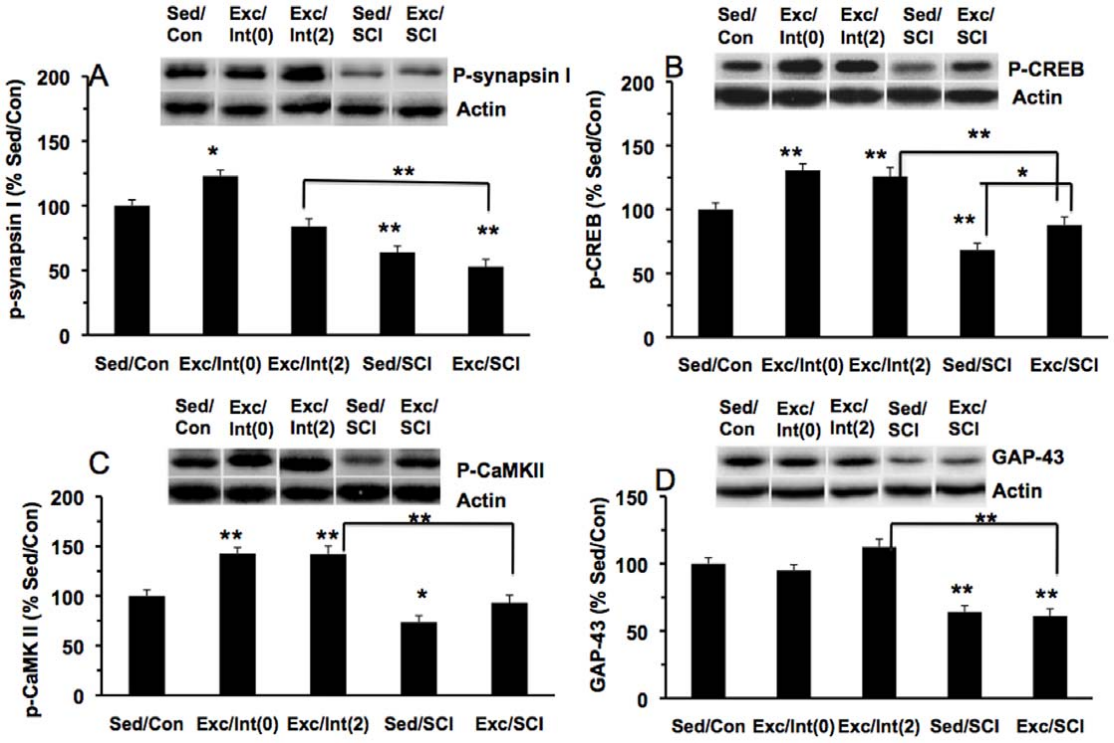
Effects of SCI and exercise on plasticity markers in the lumbar region of the spinal cord: (A) p-synapsin I; (B) p-CREB; (C) p-CaMKII; (D) GAP-43. Exercise increased protein levels of these markers in intact animals, which were sacrificed on the last exercise day (Exc/Int(0)) or two days after ceasing exercise (Exc/Int(2)). Exercise prior to SCI prevented the effects of the injury. *p<0.05, **p<0.01. Representative western blot bands are shown for each experimental group.

We measured the protein levels of the same synaptic markers in the spinal cord lumbar enlargement region as in the hippocampus. For the levels of p-synapsin I, one-way ANOVA showed a significant group effect (F(4,38) = 33.084, p<0.01). The Bonferroni post-hoc test indicated that exercise elevated the p-synapsin I levels in the Exc/Int(0) group (123%, p<0.05) while no significant changes were found in Exc/Int(2) group (Fig. 4A). SCI reduced the p-synapsin I levels to 64% (p<0.01) and 53% (p<0.01) in Sed/SCI and Exc/SCI groups, respectively (Fig. 4A).

There was a significant group difference for the levels of p-CREB (F(4,38) = 30.533, p<0.01) assessed with an one-way ANOVA (Fig. 4B). The multiple comparisons were carried out by the Bonferroni post-hoc test. The levels of p-CREB were significantly higher in the Exc/Int(0) group (131%, p<0.01) and in the Exc/Int(2) group (125%, p<0.05), as compared to the Sed/Con group. SCI reduced p-CREB levels in sedentary animals (Sed/SCI, 62%, p<0.01) and exercise prevented these effects (Exc/SCI, 88%, p>0.05).

With regards to the levels of p-CaMK II, we found a significant group difference (F(4,38) = 24.674, p<0.01) by one-way ANOVA. The Bonferroni post-hoc test performed for multiple comparisons showed that exercise increased the levels of p-CaMK II in the Exc/Int(0) group and the Exc/Int(2) group to 142% (p<0.01) and 143% (p<0.01), respectively (Fig. 4C). The levels of p-CaMK II after SCI in the sedentary group (Sed/SCI) were reduced to 74% (p<0.05), while exercise prior to injury prevented the injury reduction (Exc/SCI: 93%) such that the p-CaMK II levels remained nearby the Sed/Con animals (Fig. 4C).

For the levels of GAP-43, one-way ANOVA analysis showed a significant group difference (F(4, 38) = 19.417, p<0.01). Exercise did not change the levels of GAP-43 in the Exc/Int(0) (95%, p>0.05) and Exc/Int(2) (113%, p>0.05) animals (Fig. 4D). The levels of GAP-43 were decreased in the Sed/SCI (64%, p<0.01) and the Exc/SCI (61%, p<0.01) groups as compared to the Sed/Con group (Fig. 4D).

### Coordinated changes in BDNF in the hippocampus vs. spinal cord

**Figure 5:**
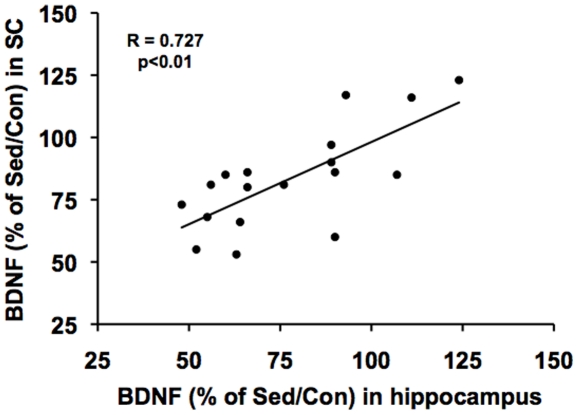
Proportional changes in the hippocampus and SC after SCI. Linear regression analysis for individual rats showed a correlation between levels of BDNF in the hippocampus Vs the SC (R = 0.727, **p<0.01). Results showed no statistical correlation for exercise and sedentary intact animals (R = 0.262, *p>0.05, data not shown).

Given the effects of the SCI on the hippocampus and SC, we sought to determine whether these effects could be coordinated. Accordingly, we performed a linear regression analysis for individual rats to evaluate the possibility of a correlation between levels of BDNF in the hippocampus Vs the SC. Results showed a high correlation in animals that had received the SCI (R = 0.727, p<0.01, Fig. 5), suggesting the possibility that the SCI coordinates a corresponding response in the brain and SC. Results showed no statistical correlation for exercise and sedentary intact animals (R = 0.262, P>0.05).

## Discussion

We have found that a complete SC transection can impact select molecular systems that support neuronal function and plasticity in the brain and SC. The fact that SCI reduced levels of BDNF and related proteins in the hippocampus indicates that the brain is also affected by the SCI pathology. Our results provide evidence for a coordinated response of the brain and spinal cord to SCI. Interestingly, exercise provided before the injury onset showed a protective action in the brain as well as in the SC as evidenced by its counteractive effects on the injury-related reductions of most of the molecular systems under study. These results emphasize the capacity of exercise to recruit molecular adaptations that can enhance plasticity in the brain and spinal cord.

### Effects of SCI on the expression of neurotrophins in the brain

Our results show that SCI reduced levels of BDNF and related plasticity markers in the hippocampus, and indicate that the SC contributes to maintain BDNF levels in the brain. In addition, based on the fact that the reduction of BDNF was accompanied by changes in proteins related to the function of BDNF on synaptic plasticity, it is assumable that these molecular adjustments have functional consequences. The studies were focused on the acute period following the injury based on the critical role of this period for injury recovery [5]. In particular, it is known that molecular activity occurring during this period is crucial for the functional capacity of the SC for learning a motorsensory task [18]. Given the active role of BDNF on synaptic plasticity, it is likely that reductions in BDNF during this period may impair synaptic transmission that is crucial for normal hippocampal function. It has recently been reported that SCI promotes neurite sprouting and elevations of synapsin I in the hindbrain of the lamprey [3]. However, there are several differences with the current study including species, timing of the effect, and brain regions involved. Changes in sprouting were observed after 1 week, which is interpreted by the authors as a compensatory reaction to the injury [3], in an animal species characterized by exuberant plasticity. In turn, in agreement with our results, Fumagalli et al [4] have reported reductions of BDNF mRNA in the hippocampus of rats 24 h after an impact induced SCI. Based on the role of the hippocampus in the processing of information important for learning and memory and emotions, our results suggest the possibility that SCI increases the vulnerability to emotional and cognitive disorders. This possibility is in agreement with clinical studies showing that SCI reduces the quality of life of patients and often is associated with increased propensity for anxiety and depression [1].

In turn, it has recently been reported that damage to the brain can also influence the brain stem and SC centers. Specifically, concussive brain injury in humans, which is known to compromise cognitive performance and other brain functions, has also been associated with gait deficits [19]. Gait deficiency likely involves multiple centers in the brain and spinal cord, and their identification requires further studies. Animal models of concussive injury, which is known to affect hippocampal-dependent cognition, have been shown to reduce levels of BDNF and associated molecules in the SC, in addition to the hippocampus [20]. Accordingly, this reciprocal interaction between the hippocampus and SC can provide a platform from which to start understanding a wide range of cognitive and motor disorders observed after SCI or brain injury.

### Effects of SCI on the expression of neurotrophins in the spinal cord

Our results showing that SCI reduced levels of BDNF in the lumbar SC region may be the results of various mechanisms. It is posible that a reduction in BDNF is caused by an interruption of suprasegmental input necessary for maintaining levels of BDNF. It is also feasibly that the injured neurons and their projections from the peri-injury region may reduce supply of BDNF. In addition, production of BDNF may be affected by the release of growth inhibitors molecules such as myelin-associated glycoproteins [21] after the injury, which are known to exert inhibitory effects on BDNF levels and function [22]. Thus, it is possible that a combination of factors may play a role in the injury-related reduction in SC BDNF plasticity.

Based on the results showing that the SCI affected levels of BDNF in the brain and SC, we sought to determine whether the injury could somehow coordinate a response in the hippocampus and SC. Accordingly, we performed linear regression analysis to correlate levels of BDNF in the brain and SC in intact and SCI animals. We found that levels of hippocampal BDNF were correlated to SC BDNF levels in animals that had received SCI, while no correlation was observed in intact animals. These results seem to emphasize the possibility that the lesion concerts a response in the brain and SC.

### Effects of exercise

Exercise elevated levels of BDNF and related molecules in the SC and hippocampus of intact animals as previously described [12], [23]. Interestingly, intact exercise animals that were allowed to rest for 2 days before the sacrifice showed higher levels of BDNF in the SC, but not in hippocampus, than those animals sacrificed immediately after exercise. These results may indicate that exercise increases axonal transport of BDNF from brain centers. Interestingly, the SCI reduced SC levels of BDNF in both the exercise and sedentary animals, suggesting that the SCI may disrupt the axonal transport. In turn, hippocampal BDNF levels in SCI animals were maintained nearby intact levels after exercise. Exercise also showed important effects in counteracting a SCI-related reduction in p-synapsin I in the hippocampus, which is in agreement with the close described association between BDNF and synapsin I [24].

The concurrent effects of exercise on the brain and spinal cord are significant to better understand the neural pathways by which exercise can affect the brain. It is known that exercise is an important promotor of BDNF-related plasticity in the hippocampus and other brain structures; however, the interaction between supraspinal and infraspinal centers during exercise remains elusive. Although running wheel exercise heavily relies on neuromuscular work, the influence of hindlimbs and rearlimbs activity on the brain has received insufficient attention. The fact that an injury at the thoraciic SC level reduced the action of exercise in hippocampal plasticity emphasizes the influence of the hindlimb activity on brain plasticity.

### Downstream BDNF effectors

To evaluate the possible functional implications for changes in the neurotrophin system resulting from SCI, we measured the levels of various molecules that have a recognized interaction with BDNF and are important for synaptic plasticity. Our results showed that SCI reduced levels of all the molecular markers examined in the hippocampus, with the exception of GAP-43. These results emphasize the importance of the SC activity on maintaining brain function and plasticity, i.e, BDNF and synaptic markers in the hippocampus. Exercise showed a clear elevation in p-CREB, p-CaMKII, and GAP-43 in the hippocampus of intact rodents, and the SCI counteracted the effects of exercise. It is interesting that changes in p-synapsin I followed closely the BDNF pattern in the hippocampus. The coordinated response between BDNF and synapsin I may be explained by the established association between these two molecular systems in the hippocampus. BDNF phosphorylates synapsin I to induce the mitogen-activated protein kinase signaling pathway which, in turn, modulates neurotransmitter release [14]. In addition, we have been able to reduce the exercise-induced increase in synapsin I mRNA in the hippocampus by blocking BDNF action using the tyrosine kinase receptor blocker K252A [23]. Therefore, the change in synapsin I in the present study is a likely indicator of modified synaptic plasticity associated to the function of BDNF.

Similar to BDNF, synapsin I levels were down-regulated in the SC following SCI. Exercise was not sufficient to maintain levels of p-synapsin I elevated in the SC of SCI rats, which suggests that the 2-day rest period may have weaken the effects of exercise. Indeed, exercise was able to elevate levels of p-synapsin I in intact animals at day 0 but not after the 2-day rest period. Exercise in the SC was effective in counteracting a reduction in p-CREB.

### Conclusions and functional implications

Results emphasize the impact of SCI on molecular systems that support neuronal function and plasticity in the brain and spinal cord. In particular, exercise was able to counteract the injury-related reductions in BDNF, CaMKII, and CREB in the hippocampus, and exercise prior the injury appeared to normalize these reductions. These findings suggest that an active lifestyle may confer the CNS with the capacity to defend against neurological and cognitive weaknesses after SCI. In particular, our results may be significant to understand the influence of pre-injury conditions such as lifestyle factors on the capacity of patients for healing following neurological damage. The fact that pre-injury conditions can have an impact on the degree of recovery may also help explain the high degree of individual variability in the patient response to treatment.

## Materials and Methods

The experiments were performed in accordance with the United States National Institutes of Health Guide for the Care and Use of Laboratory Animals. All procedures were approved by the UCLA Chancellor's Animal Research Committee.

### Exercise paradigm

**Figure 6:**
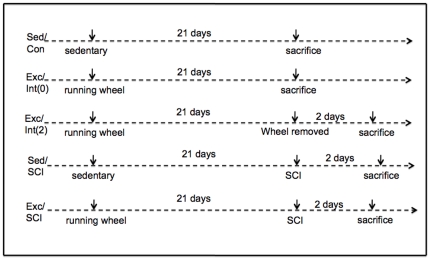
Schematic diagram of the experimental design showing the timing for the applications of exercise (Exc) and spinal cord injury (SCI) in the various experimental groups.

The experiments were performed in male C57BL/6 mice (Charles River Laboratories International, Inc.). The mice approximately 10 weeks of age were housed individually in standard polyethylene cages in an environmentally controlled room (22–24°C) with a 12 h light/dark cycle. Animals were either exposed to cages provided with running wheel (exercise) or regular cages (sedentary). The cages equipped with running wheels (diameter = 12 cm, width = 5 cm, Mini Mitter, Bend, Oregon, USA) allowed animals to exercise *ad libitum*. Running revolutions were recorded using VitalViewer software (Mini Mitter, Bend, Oregon, USA). After completing a 3-week period, exercise animals were divided into three groups: animals sacrificed the morning of the last day of exercise (Exc/Int(0)); animals sacrificed 2 days after the last exposure to exercise (Exc/Int(2)); animals received a full transection lesion into the SC and sacrificed two days after the injury (Exc/SCI) (Fig. 6). Sedentary rats received a full transection lesion into the SC and were sacrificed two days later (Sed/SCI) (Fig. 6). Sedentary animals without injury were used as the main control group (Sed/Con) (Fig. 6). The average running distance between three groups was not significantly different (Exc/Int(0):8.63±0.36 km/day; Exc/Int(2): 8.69±0.69 km/day; Exc/SCI: 8.99±0.49 km/day).

### Lesion

Briefly, the mice were pre-sedated with buprenorphine (0.05 mg/kg body weight, s.c.) and mice were maintained in a deep anesthetic state throughout the surgery with isoflurane gas (1.5–2.5% isoflurane mixed with 100% O2 at a flow rate of .4 L/minute via face mask). A complete SC transection at a mid-thoracic level (T7–T9) was performed under aseptic conditions. A laminectomy was made in the vertebral column in the mid-thoracic region (∼T7–T9), the dura opened and the SC exposed. The spinal cord was lifted with a curved probe and completely transected at the T8–T9 junction. The surgical areas were lavaged and wounds closed with sutures. All animals were continually monitored until the tissue collection time.

### Tissue preparation and protein determination

Animals were sacrificed in the early morning. Brain and SC tissues were dissected, frozen on dry ice, and stored at −70°C until further use. Hippocampus and SC lumbar enlargement regions were homogenized in a freshly prepared lysis buffer (137 mM NaCl, 20 mM Tris-HCl pH 8.0, 1% NP-40, 10% glycerol, 1 mM phenylmethylsulfonyl fluoride, 10 µg/mL aprotinin, 1 µg/mL leupeptin, 0.5 mM sodium vanadate). Homogenates were centrifuged at 12,000 g for 20 min to remove insoluble material. The supernatants were collected into clean 1.5 ml tubes and stored at −70°C. The total protein concentration of homogenates was determined with a MicroBCA kit (Pierce, Rockford, IL, USA), using BSA as a standard.

### Western blot

A total of 25 µg of protein from each sample was used for western blot experiment. Sample proteins were separated on 10% SDS-polyacrylamide gels. After electrophoresis, the proteins were transferred onto PVDF membranes and nonspecific binding was blocked with 5% nonfat dry milk in Tris-buffered saline containing 0.05% Tween-20 (TBST).The membranes were incubated with a primary antibody overnight at 4°C, followed by a secondary antibody for 1 hr at room temperature. The following primary antibodies were used: BDNF (1∶1000, Santa Cruz Biotechnology), phospho-synapsin I (1∶1000, Cell Signaling), phospho-CREB (1∶1000, Millipore), phospho CaMKII (1∶1000, Santa Cruz Biotechnology), GAP-43 and actin (1∶1000, Santa Cruz Biotechnology). The secondary antibodies used were anti-goat or anti-rabbit IgGHRP (1∶10,000, Santa Cruz Biotechnology). After rinsing with buffer (0.1% Tween-20 in TBS), the immunocomplexes were visualized by chemiluminescence using the Amersham ECL Plus Western Blotting Detection kit (GE Healthcare Bio-Sciences, Piscataway, NJ, USA) according to the manufacturer's instructions. The film signals were digitally scanned using a HP Scanner (HP Scanjet 3970) and quantified with NIH Image software. Actin was utilized as an internal control for sample loading, and each blot was normalized to its corresponding actin value.

### Immunohistochemistry

Fixed brain tissues (n = 4/group; 4% w/v paraformaldehyde) were sliced in the sagittal plane (25 um) and spinal cord tissues (n = 4/group) were sliced in the transverse plane (25 um), collected free floating in PBS, and processed for BDNF immunohistochemistry, as previously described [25]. A 1∶1,000 dilution was used for the rabbit polyclonal anti-BDNF antisera (BDNF antibody was purchased from Millipore). Immunostained sections were visualized using a Zeiss Axio Imager HI microscope with the ApoTome Slider Axioimaging system.

### Statistical analyses

A total of 43 mice were used for western blot assays (Sed/Con: n = 10; Exc/Int(0): n = 10; Exc/Int(2): n = 5; Sed/Sci: n = 11; Exc/SCI: n = 7). Results of western blot analysis were expressed as a mean percent of intact animals maintained under sedentary conditions (Sed/Con) ± standard error of the mean (SEM). We had two groups of intact animals that were exposed to exercise: animals sacrificed the morning of the last day of exercise (Exc/Int(0)), and animals sacrificed 2 days after the last exposure to exercise (Exc/Int(2)). The rationale for the Exc/Int(2) group was to provide a reference point to evaluate the effects of the two inactive days between the last day of exercise and sacrifice.

All statistical analyses were done by statistic software SPSS 16.0. Protein data were analyzed by one-way ANOVA. Post hoc tests were conducted using Bonferroni comparisons. A linear regression analysis was performed to assess correlation between levels of BDNF in hippocampus Vs SC. Group differences were considered significant when p<0.05.
